# Case report: Spontaneous remission in lung carcinoma with a late relapse after adjuvant immunotherapy: Exceptional tumor micro-environment

**DOI:** 10.3389/fimmu.2023.1106090

**Published:** 2023-02-27

**Authors:** Yan Chen, Wenhui Guan, Changhao Zhong, Jiaxi Deng, Minjuan Hu, Wenwei Mo, Xiaohong Xie, Shiyue Li, Chengzhi Zhou, Xinqing Lin

**Affiliations:** Pulmonary and Critical Care Medicine, Guangzhou Institute of Respiratory Health, National Clinical Research Center for Respiratory Disease, National Center for Respiratory Medicine, State Key Laboratory of Respiratory Diseases, The First Affiliated Hospital of Guangzhou Medical University, Guangzhou, China

**Keywords:** immune checkpoint inhibitors, spontaneous remission, tumor immune microenvironment, non-small cell lung cancer, adjuvant immunotherapy

## Abstract

Spontaneous remission (SR) of local recurrence after adjuvant immunotherapy has rarely been reported, and the underlying mechanism is poorly understood. Herein, we reported a patient with stage cT2aN2M0 squamous cell lung carcinoma who received neoadjuvant and adjuvant treatment with nivolumab plus chemotherapy. The patient experienced a late relapse in the subcarinal lymph node seven months after the last dosage of treatment but achieved SR in the next three months without additional antitumor therapy. The complete response lasted for eleven months and counting. Notably, high copies of pathogenic microorganisms were detected in the patient’s bronchoalveolar lavage fluid along with the recurrence but disappeared after SR. The patient also experienced a lymph node puncture-induced fever but had no other symptoms. A longitudinal analysis of infiltrated immune cells in the recurrent lymph node was performed by multiplex immunofluorescence and whole transcriptome sequencing, which revealed that CD8+ T cells were recruited during the initial relapse, specifically in the stromal area, then migrated into the tumor tissue, and continued to increase after elimination of tumor cells. Meanwhile, the initial recruitment of CD8+ T cells was coupled with a higher proportion of B cells, and the abundant neutrophil population was synchronous with the infiltration of CD8+ T cells into tumor cells. This is the first report on an Non-small cell lung cancer (NSCLC) patient with a late relapse after adjuvant immune checkpoint inhibitor (ICI) therapy who achieved SR. Our case highlights the complexity and plasticity of antitumor immunity and is expected to help find efficient strategies against the resistance of ICI treatment.

## Introduction

Immune checkpoint inhibitors (ICIs), such as programmed cell death protein (PD-1) and programmed death-ligand 1 (PD-L1) inhibitors, have demonstrated impressive long-term survival benefits and have been approved as first-line treatment for advanced non-small cell lung carcinoma (NSCLC) as well as neoadjuvant/adjuvant therapy for resectable settings, either as monotherapy or combined with chemotherapy (1–3). Despite unprecedented and durable clinical efficacy, ICI treatment could lead to complex and heterogeneous patterns of response and resistance that lack consensus definitions (4). The biology underlying these patterns remains elusive and better understanding is needed to determine coping strategies.

Here, we presented a case with resectable squamous cell lung carcinoma who underwent neoadjuvant and adjuvant treatment with nivolumab plus chemotherapy, followed by maintenance therapy with nivolumab monotherapy. The patient experienced a lymph node recurrence resembling the late-relapse pattern (defined as progressive disease (PD) ≥ 12 weeks after the last dosage of adjuvant treatment (4)) and achieved spontaneous remission without additional anti-cancer treatment. Changes in the tumor immune microenvironment (TIME) during the course were evaluated by whole transcriptome sequencing(WTS) and multiplex immunofluorescence (mIF) analyses.

## Case presentation

**Figure 1:**
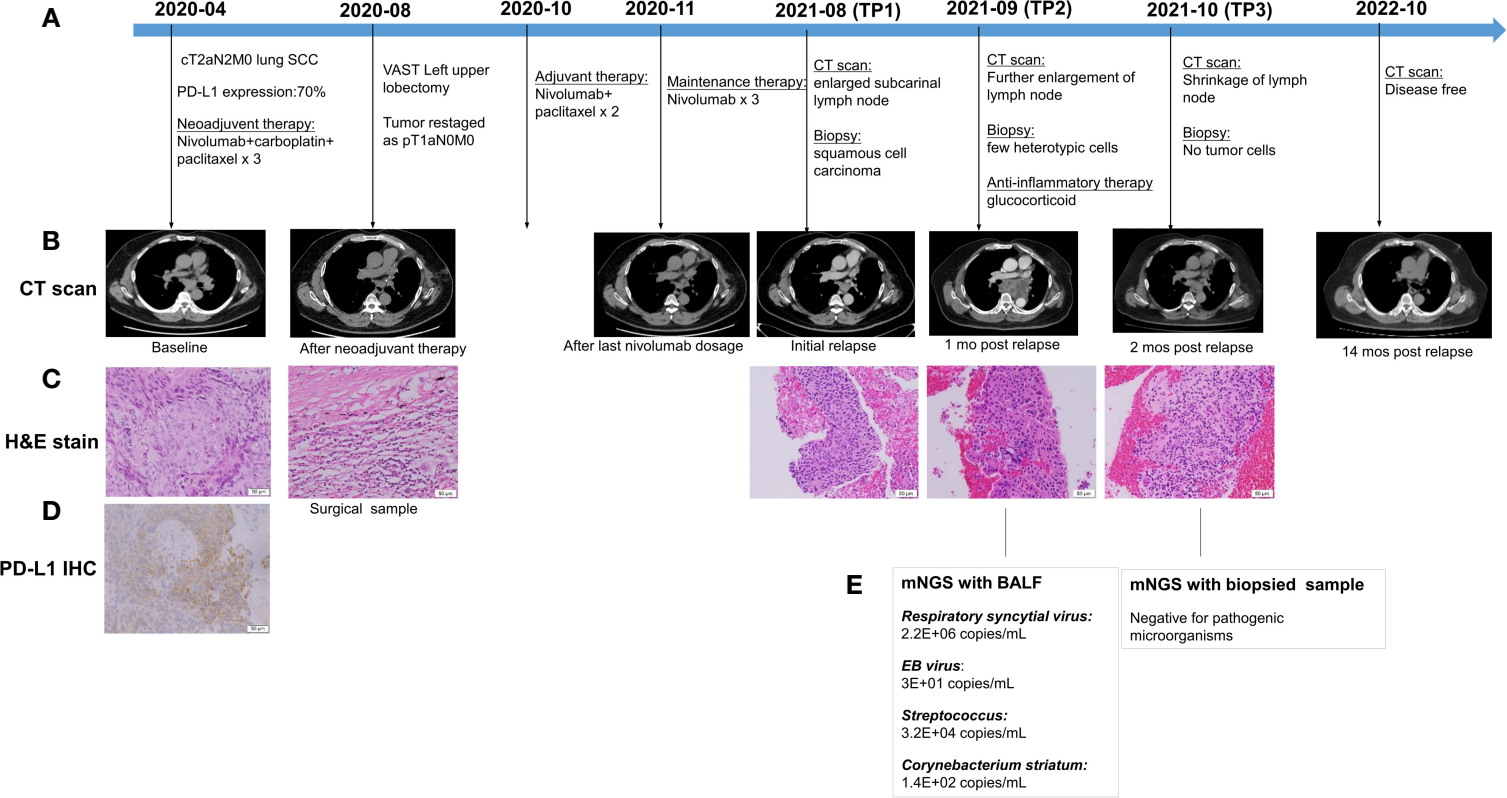
The clinical course of the patient **(A)** Timeline showing the clinical course; **(B)** Change of the tumor lesion revealed by CT scan; **(C)** Hematoxylin and Eosin (H&E) stain of biopsied and surgical samples; **(D)** PD-L1 expression assessed by immunohistochemistry (IHC) assay; **(E)** Results of metagenomic next-generation sequencing (mNGS) with patient’s bronchoalveolar lavage fluid (BALF) and biopsied sample. TP1: initial relapse; TP2: 1 month after relapse; TP3: 2 months after relapse.

A 55-year-old male with a smoking history of 40 pack/year visited our clinic in April 2020 due to a mass (46×22 mm) in the left upper lobe revealed by a chest CT scan (**Figures 1A, B**). The patient presented with occasional cough and white phlegm, and had an unremarkable medical history. An endobronchial ultrasound (EBUS) guided transbronchial biopsy was performed, and the histopathological examination revealed lung keratinizing squamous cell carcinoma (**Figure 1C**) with strong positive PD-L1(22C3) expression (70%) (**Figure 1D**). There was no evidence of metastases. Enlarged ipsilateral hilar and mediastinal lymph nodes were detected, and a stage of cT2aN2M0 was determined. The patient received three cycles of neoadjuvant treatment with nivolumab (200mg, q3w) combined with carboplatin (350mg, q3w) plus paclitaxel (300mg, q3w). After neoadjuvant immunotherapy, a chest CT showed that the mediastinal and left hilar lymph nodes shrank (**Figure 1B**), so the patient underwent a left upper lobectomy in August 2020. The maximum diameter of the resected tumor was 1.4 cm. No viable tumor cells were found in the resection (necrotic tissue 65%; inflammatory cells and fibrous tissue 35%) (**Figure 1C**) and no cancer cells were detected in lymph nodes. The stage was revised to pT1aN0M0. Postoperatively, the patient received two cycles of nivolumab (200mg, q3w) plus paclitaxel (300mg, q3w), followed by three cycles of maintenance therapy with nivolumab monotherapy (200mg, q3w), and remained disease-free (**Figure 1B**).

However, seven months after the completion of maintenance therapy, a follow-up CT scan revealed an enlarged subcarinal lymph node measuring 2.2 cm (**Figure 1B**). Lymph node biopsy *via* bronchoscopy showed squamous cell carcinoma (**Figure 1C**). Radiotherapy was initially planned but postponed due to the patient’s fever. After one month, a repeated chest CT scan showed fusion, further enlargement of the subcarinal lymph node (measuring 10.6cm), and circular low-density shadow with a small cavity, indicating lymph node recurrence but with partial tumor necrosis. (**Figure 1B**). Only a few atypical cells were found in the biopsied sample of the lymph node (**Figure 1C**).

Meanwhile, high copies of respiratory syncytial viruses (RSV), EB virus, Streptococcus, Corynebacterium striatum, and other microorganisms were detected in the patient’s bronchoalveolar lavage fluid by the metagenomic next-generation sequencing (mNGS) technique (**Figure 1E**) using the MAPMI™ panel on BioelectronSeq 4000 (CapialBio, Beijing, China). The patient experienced a fever of up to 40°C after lymph node puncture but had no other symptoms, suggestive of an immune-mediated inflammatory response. He subsequently received glucocorticoid therapy. One month later, a chest CT revealed marked shrinkage of the enlarged lymph node (2.8cm in diameter) (**Figure 1B**). No tumor cells were found with the bronchial lymph node biopsy. Only a small amount of moderate atypical hyperplasia of squamous epithelial cells and granulation tissue were observed (**Figure 1C**). mNGS test with the biopsied sample was negative for pathogenic microorganisms (**Figure 1E**).

**Figure 2:**
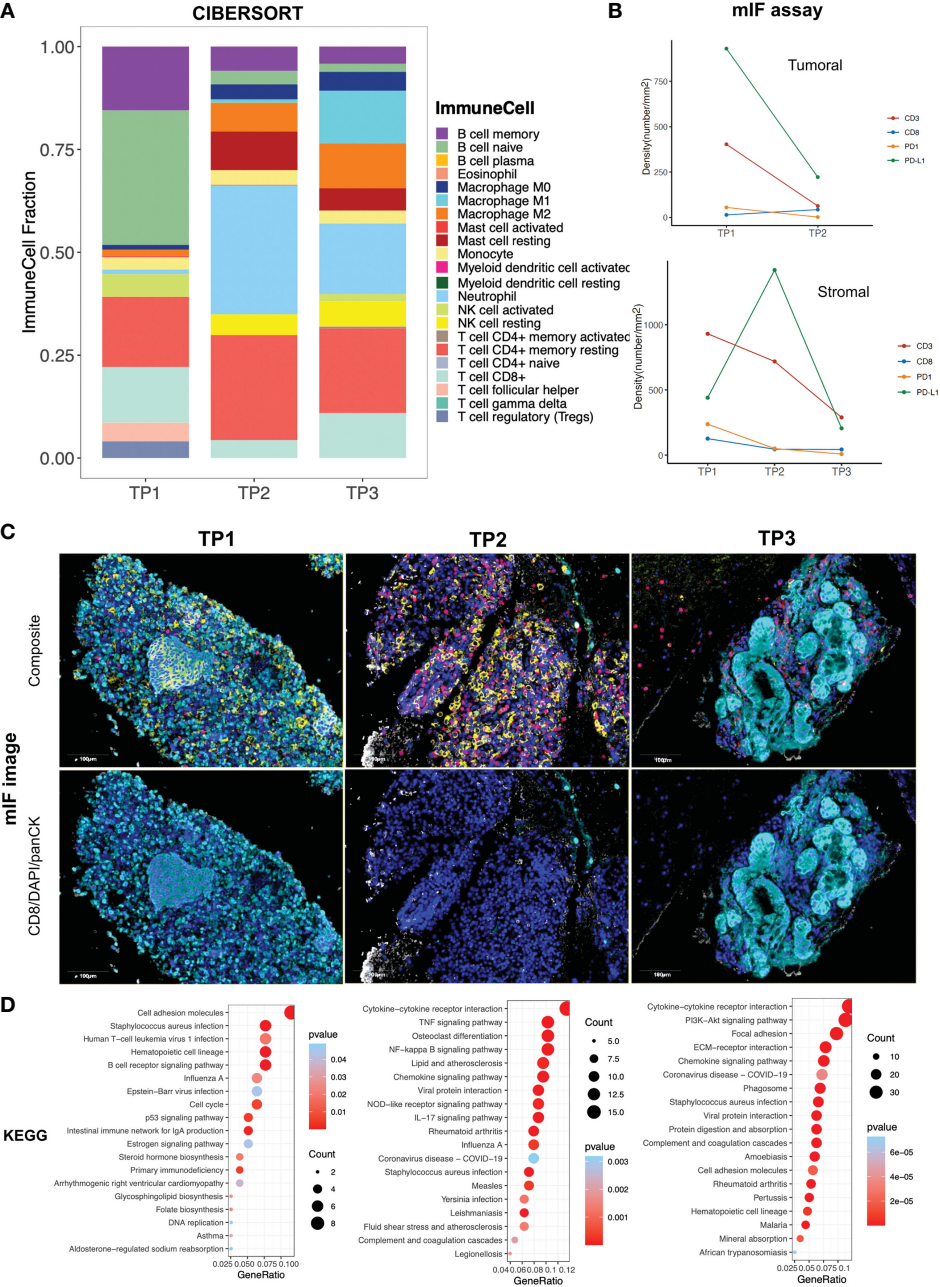
The dynamic change of tumor microenvironment. **(A)** Proportions of various infiltrated immune cells estimated by CIBERSORT based on whole transcriptome sequencing (WTS) data; **(B)** Densities of different multiplex immunofluorescence (mIF) markers assessed by inForm image analysis software (PerkinElmer, Waltham, Massachusetts, US); **(C)** Multiple immunoflorescent stain captured by Mantra System (PerkinElmer, Waltham, Massachusetts, US), composite five-color images showing the expression of PD-1(green), PD-L1(yellow), CD3 (red), CD8 (white), and panCK (cyan) DAPI(blue) and images showing CD8, DAPI, and panCK markers; **(D)** KEGG pathway enrichment. TP1: initial relapse; TP2: 1 month after relapse; TP3: 2 months after relapse.

WTS and mIF were performed on formalin-fixed paraffin-embedded (FFPE) blocks from the enlarged lymph node to evaluate the tumor immune microenvironment (TIME) change during this exceptional scenario. The proportion of tumor cells declined from 10% at the initial relapse (TP1) to 2% one month later(TP2) and dropped to 0% two months after relapse (TP3) as assessed by an independent pathologist. CIBERSORT analysis based on the WTS data (5) revealed that the proportion of CD8+ T cells was the highest at TP1, decreased at TP2, and increased again at TP3 (**Figure 2A**). Moreover, a high proportion of B cells (both memory and naïve) and neutrophils was estimated at TP1 and TP2, respectively. The proportion of M1 macrophages was upregulated at TP3. Regulatory T cells (Tregs) were present only at TP1. The mIF panel includes markers for CD3+, CD8+, PD-1, PD-L1, and panCK (**Table S1**) (6). The results showed an increase of CD8+ T cell density in the tumoral compartment from TP1 (14 no./mm^2^) to TP2 (43 no./mm^2^) (**Figures 2B, C**) (**Table S2**). Comparatively, in the stromal compartment, CD8+ T cell density decreased from TP1 (127 no./mm^2^) to TP2 (46 no./mm^2^) and then remained unchanged at TP3 (44 no./mm^2^). The densities for CD3+ and PD1+ cells dropped both in the tumoral (from TP1 to TP2, CD3+: 403 to 63 no./mm^2^, PD1+: 55 to 2 no./mm^2^) and stromal (TP1, TP2, TP3, CD3+: 931, 719, 290 no./mm^2^, PD1+: 238, 51, 9 no./mm^2^) compartments. PD-L1+ cell density deceased from TP1 (930 no./mm^2^) to TP2 (222 no./mm^2^) in the tumor area, but increased and then declined in the stromal area (TP1:441no./mm^2^, TP2:1420 no./mm^2^, TP3:206 no./mm^2^). We further explored differential gene expression at the three time points by Kyoto Encyclopedia of Genes and Genomes (KEGG) term enrichment analysis. The results indicated an enrichment of the B cell receptor signaling pathway at TP1. Cytokine to cytokine receptor interaction and chemokine signaling pathway were enriched both at TP2 and TP3. Besides, enrichments of NF-kappa B and TNF signaling pathways were noted at TP2 (**Figure 2D**).

Of note, the patient maintained sustained control without additional antitumor treatment until the last follow-up in October 2022 (**Figure 1B**), and no adverse event was observed.

## Discussion and conclusion

The Society for Immunotherapy of Cancer (SITC) has defined the late-relapse pattern as a PD ≥ 12 weeks after the last dose of adjuvant therapy with ICI to discriminate it from a relapse considered as primary resistance, which emerges < 12 weeks after adjuvant therapy (4). Herein, we reported a rare case with NSCLC who achieved spontaneous and sustained remission after experiencing a late relapse seven months after discontinuing adjuvant therapy with nivolumab. Spontaneous remission rarely occurs in clinical situations, which is known as “the partial or complete resolution of the malignant tumor with no or inadequate antitumor treatment” (7). Spontaneous remission of lung cancer is even more scarce, accounting for no more than 2.6% of all cases (8). Only sporadic NSCLC cases confirmed by biopsy have been reported (9). The mechanism underlying this scenario has yet to be fully elucidated, but more likely due to the change of the tumor microenvironment (TME).

CD8+ T lymphocytes are pivotal tumor-killing immune cells in cancer treatment with ICIs and are usually short-lived. After tumor cells are eliminated, most of the CD8+ T cells will die. Still, a small population of them remains in the form of long-lived memory T cells, which are self-renewing and can generate cytotoxic CD8+ T cells if malignancy is reencountered (10). In our case, we observed that CD8+ T cells accumulated in the stroma area at the initial relapse (TP1), then migrated into the tumoral compartment (TP2), and continued to be recruited in the stroma area after elimination of tumor cells (TP3) (**Figures 2B, C**). It was speculated that the initial relapse might be attributed to the dormancy of residual molecular disease, impaired T-cell activation (loss of antigen presentation, etc.), or/and inaccessibility of T cells into tumor cells (4, 11). In response to the emergence of tumor cells, CD8+ T cells were promptly re-primed/activated/awakened. Of note, WTS indicated enrichment of B cells at TP1 (**Figures 2A, D**), which supported the speculation of T cell activation at this time point, given that B cells can exert antitumor immunity by enhancing tumor-associated antigen presentation to proximal T cells (12, 13).

At TP2, an enrichment of the chemokine signaling pathway was noted. Chemokines direct the migration of immune cells in tumor tissues (14). This observation concorded with the increased infiltration of CD8+ T cells in the tumor area at TP2. Moreover, an abundant population of neutrophils was recruited to relapse sites at TP2, presenting with rapidly growing lymph nodes. Despite dual functions in the tumor, the antitumor properties of neutrophils are manifested as the direct killing of cancer cells and/or producing accessory signals (chemokine, for instance) to activate T cells. Meanwhile, B cells in peripheral blood first increased and then decreased to a normal range, while T cells decreased significantly and returned to normal. Collectively, the results revealed an active antitumor TME at TP2.

Continuing upregulation of the chemokine signaling pathway and recruitment of CD8+ T cells were observed in relapsed lymph nodes when no visible tumor lesions were detected (TP3). This observation suggested that an immune surveillance status was maintained at this time point, conferring the sustained antitumor immunity of the patient.

**Figure 3:**
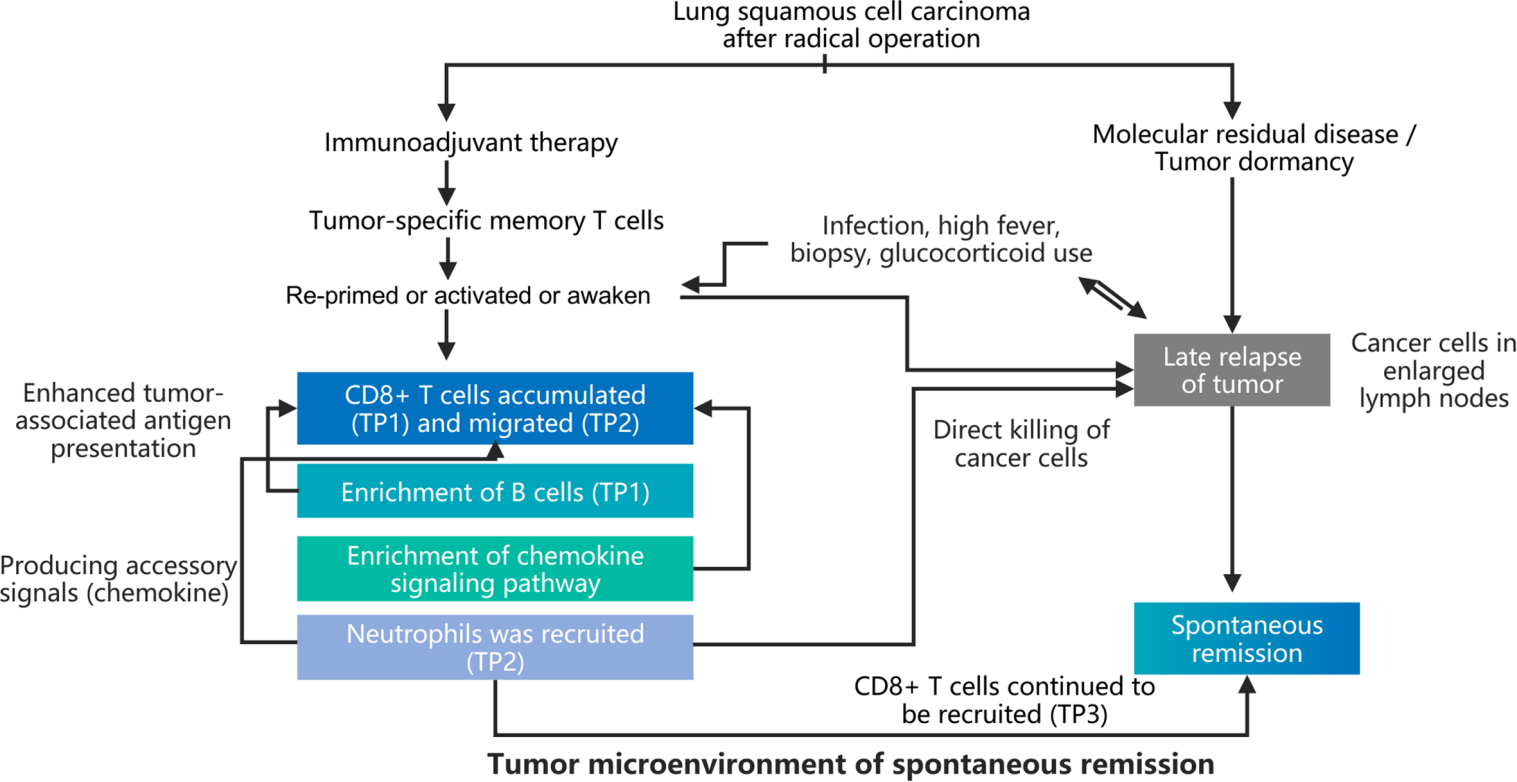
Diagram illustrating the potential mechanisms underlying the spontaneous remission.

Intriguingly, we also observed high copies of pathogenic microorganisms such as RSV and EB virus accompanied by lymph node recurrence, which disappeared after spontaneous remission. It was speculated that microbial products and microbial-related immune cells were involved in the spontaneous tumor remission. However, our detection technique could not effectively distinguish tumor-specific T cells from microbiota-specific T cells. Sporadic case reports have described spontaneous tumor remission induced by infection with severe fever (15), the use of corticosteroids, or puncture biopsy (16). Still, the underlying mechanism has not been fully elucidated in these studies. In our case, the adjuvant immunotherapy, infection-mediated immune system activation, and biopsy procedures have a synergistic effect on the tumor microenvironment (**Figure 3**).

Our study was the first to decipher the dynamic TME change during spontaneous tumor remission. However, as mentioned above, our study could not identify tumor/microbiota-specific immune cells. Future studies with T-cell receptor (TCR)/B-cell receptor (BCR) sequencing may shed light on the complex interplay mechanism. Moreover, TME analysis on the pretreated and resected primary lesions will complete the evolutionary story.

To the best of our knowledge, this was the first case in which an NSCLC patient with a late relapse achieved spontaneous remission after ICI withdrawal. It has been suggested that patients with PD >12 weeks after stopping ICI treatment may consider the therapeutic rechallenge (4). Our case revealed an exceptional clinical scenario where the rechallenge with ICI may not be necessary and underscores the importance of confirming the recurrent disease by biopsy in the adjuvant setting of NSCLC. Our results also depicted the dynamic alteration in TME during this process, highlighting the complexity and plasticity of antitumor immunity in response to ICI treatment and leading us to wonder whether “inducing” spontaneous tumor remission could be a novel and efficient strategy against immunotherapy resistance.

## Data availability statement

The original contributions presented in the study are included in the article/**Supplementary Material**. Further inquiries can be directed to the corresponding authors.

## Ethics statement

The studies involving human participants were reviewed and approved by the First Affiliated Hospital of Guangzhou Medical University. The patients/participants provided their written informed consent to participate in this study. Written informed consent was obtained from the individual(s) for the publication of any potentially identifiable images or data included in this article.

## Author contributions

YC, WG, and XL contributed to the conceptualization; YC and CHZ contributed to the data analysis; JD, MH, WM, and SL contributed to the experiment; CZZ and XL contributed to the resource; YC drafted the manuscript. WG and XL make substantial contribution to manuscript revision. All authors contributed to the article and approved the submitted version.
