# Supplementary material for: Case report: Spontaneous remission in lung carcinoma with a late relapse after adjuvant immunotherapy: Exceptional tumor micro-environment
Source: Front Immunol. 2023 Feb 27;14:1106090. doi: 10.3389/fimmu.2023.1106090 (PMC10008903; doi:10.3389/fimmu.2023.1106090)
Supplement: Supplementary file 1 [file Table_1.docx]

**Table S1.** Details of markers used in the multiplex immunofluorescence assay

|  | Clone | Vendor | Cat# | Dilution | Fluorophores |
| --- | --- | --- | --- | --- | --- |
| PD-L1 | E1L3N | CST | CST13684 | 1:300 | PPD650 |
| PD-1 | D4W2J | CST | CST86163 | 1:500 | PPD520 |
| CD3 | SP7 | Abcam | Ab16669 | 1:800 | PPD570 |
| CD8 | C8/144B | CST | CST70306 | 1:100 | PPD480 |
| Pan-CK | C11 | CST | CST4545 | 1:100 | PPD780 |

**Table S2.** Densities of different markers.

|  | Density (No./mm^2^) | | | | | |
| --- | --- | --- | --- | --- | --- | --- |
|  | Tumoral area | | | Stromal area | | |
| Marker | TP1 | TP2 | TP3 | TP1 | TP2 | TP3 |
| PD-L1 | 930 | 222 | NA | 441 | 1420 | 206 |
| PD-1 | 55 | 2 | NA | 238 | 51 | 9 |
| CD3 | 403 | 63 | NA | 931 | 719 | 290 |
| CD8 | 14 | 43 | NA | 127 | 46 | 44 |
